# Gold(III) Complexes: An Overview on Their Kinetics, Interactions With DNA/BSA, Cytotoxic Activity, and Computational Calculations

**DOI:** 10.3389/fchem.2020.00379

**Published:** 2020-05-20

**Authors:** Snežana Radisavljević, Biljana Petrović

**Affiliations:** Department of Chemistry, Faculty of Science, University of Kragujevac, Kragujevac, Serbia

**Keywords:** gold(III) complexes, DNA, bovine serum albumin (BSA), kinetics, cytotoxicity, computational calculations, density functional theory (DFT) calculations

## Abstract

In the last few years, metallodrugs play a key role in the development of medicinal chemistry. The choice of metal ion, its oxidation state and stability, and the choice of inert and labile ligands are just some of the very important facts which must be considered before starting the synthesis of complexes with utilization in medicinal purpose. As a result, a lot of compounds of different transition metal ions found application for diagnostic and therapeutic purpose. Beside all, gold compounds have attracted particular attention. It is well-known that gold compounds could be used for the treatment of cancer, HIV, rheumatoid arthritis (chrysotherapy), and other diseases. This metal ion has unoccupied d-sublevels and possibility to form compounds with different oxidation states, from −1 to +5. However, gold(I) and gold(III) complexes are dominant in chemistry and medicine. Especially, gold(III) complexes are of great interest due to their structural similarity with cisplatin. Accordingly, this review summarizes the chemistry of some mononuclear and polynuclear gold(III) complexes. Special attention is given to gold(III) complexes with nitrogen-donor inert ligands (aliphatic or aromatic that have a possibility to stabilize complex) and their kinetic behavior toward different biologically relevant nucleophiles, mechanism of interaction with DNA/bovine serum albumin (BSA), cytotoxic activity, as well as computational calculations.

## Introduction

It is already known that cancer is a huge global problem (Abdnoor and Albdali, 2019). Due to that fact, discovery and utilization of different anticancer agents are the most investigated field. The first metal-based anticancer agent was cisplatin, discovered in 1969 (Rosenberg et al., 1969). After application of cisplatin, other platinum(II) complexes (carboplatin, oxaliplatin) were employed with the same aim (Sankarganesh et al., 2019). The promising results of usage of platinum complexes in medicinal purpose have led to the synthesis of different transition metal ion compounds and study of their potential antitumor activity (Jung and Lippard, 2007; Kim et al., 2008; Garcia et al., 2009; Wu et al., 2009). The most common are complexes of copper, vanadium, ruthenium (Lazarević et al., 2017; Leon et al., 2017), rhodium (Chen et al., 2017), nickel, palladium (Jahromi et al., 2016), iron, cobalt, gold (Ott and Gust, 2007; Romero-Canelón and Sadler, 2013), and others. But, only a few of them, such as complexes of palladium(II), nickel(II), and copper(II), represent promising candidates for the treatment of cancer (Al-Masoudi et al., 2010; Andrew and Ajibade, 2018; Malik et al., 2018; Abdnoor and Albdali, 2019), while some of them, such as metalloid compound arsenic trioxide, are approved (Park et al., 2000; Miller et al., 2002). Namely, published results confirm the fact that metal complexes represent an arsenal with a wide and fundamental potential against cancer than small organic molecules (Luo et al., 2014; Rescifina et al., 2014; Alafeefy et al., 2015) due to possibility to change their properties with the right choice of oxidation state. Moreover, the choice of ligand has great impact on solubility, reactivity, and biological activity as well (Savić et al., 2016).

Chrysotherapy is closely connected with usage of gold(I) compounds in medicine, and it is known from ancient time (Sadler and Sue, 1994; Pricker, 1996; Shaw, 1999). The beginning of the twentieth century was very important for “gold chemistry” due to the discovery of Koch connected with utilization of potassium gold(I)-cyanide like anti-tuberculosis agent (Sadler, 1976). Almost at the same time, Forestier discovered usage of gold(I) complexes in the treatment of rheumatoid arthritis (Pope et al., 2002). At the end of the twentieth century, Food and Drug Administration allowed utilization of auranofin, a gold(I) complex, as oral anti-arthritic drug (Chaffman et al., 1984). Besides auranofin, other gold(I) compounds, such as aurothiomalate or aurothioglucose, were used like anti-arthritic agents. Further investigation in this field included the study of some gold(III) compounds with promising anti-inflammatory, antiparasitic, and anticancer activities (Navarro, 2009; Ott, 2009; Travnicek et al., 2012; Bertrand and Casini, 2014; Giorgio and Merlino, 2020). The results show that the primary modes of action for gold complexes are interactions with cysteine or selenocysteine containing various enzymes (tioredoxin reductase, phosphatases, cathepsin) (Roder and Thomson, 2015), while some of them undergo autophagy or interaction with topoisomerase I or ubiquitin–proteasome system (Milacic and Dou, 2009; Soave et al., 2017).

Considering that gold(III) ion forms complexes with square-planar geometry, similar to the structure of cisplatin, a number of different gold(III) complexes were studied as potential antitumor drugs. The published results confirm that these complexes can act against tumors which are resistant to cisplatin treatment as well as some of them give significant values for *in vitro* and *in vivo* cytotoxicity (μM) against solid cancer tumors without systemic toxicity (Sun and Che, 2009; Ronconi et al., 2010; Nordon et al., 2014; Huang et al., 2018; Wang et al., 2019).

Also, many scientists revealed that gold(III) complexes have great potential in the prevention of cancer (Bertrand and Casini, 2014), HIV, bronchial asthma, and like antimicrobial agents (Ott, 2009; Glišić and Duran, 2014). But, very fast hydrolysis and reduction to Au(I) or Au(0) indicate the lability of gold(III) complexes. On the other hand, their promising stability can be reached by the careful choice of appropriate inert ligands (such as polydentate ligands with sulfur, oxygen, or nitrogen as donor atoms) (Ott and Gust, 2007). Literature evidences the DNA-independent activation of some gold(III) complexes, while for some of them were proved binding to DNA, leading to the potential cytotoxic activities (Patel et al., 2013). Based on the previously mentioned, we have decided to write this review that cover interactions of novel gold(III) complexes with small biomolecules, DNA, model proteins, such as bovine serum albumin (BSA), their kinetic properties, and biological activity supported by computational calculations.

## Biological Targets for Gold(III) Complexes

**Figure 1:**
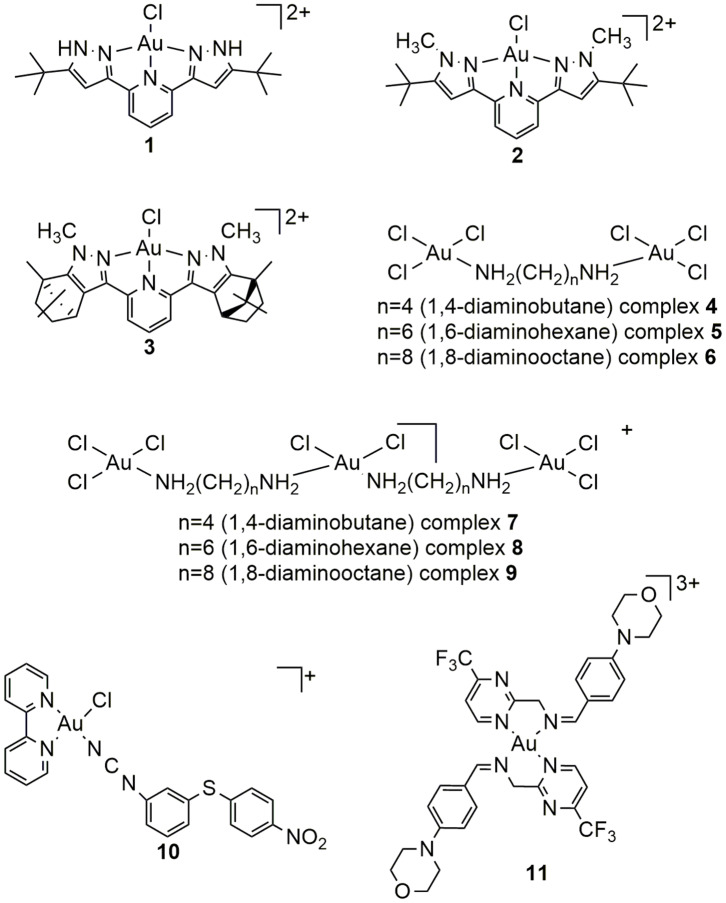
Structures of gold(III) complexes with aromatic or aliphatic nitrogen-donor ligands.

The interactions between newly synthesized gold(III) complexes that contain structurally distinct nitrogen-donor ligands (Figure 1), such as 2,6-*bis*(5-*tert*-butyl-1H-pyrazol-3-yl)pyridine (complex **1)**, 2,6-*bis*(5-*tert*-butyl-1-methyl-1H-pyrazol-3-yl)pyridine (complex **2)**, 2,6-*bis*((4S, 7R)−1,7,8,8-tetramethyl-4,5,6,7-tetrahydro-1H-4,7-methanoindazol-3-yl)pyridine (complex **3)**, 1,4-diaminobutane (complexes **4** and **7)**, 1,6-diaminohexane (complexes **5** and **8)**, 1,8-diaminooctane (complexes **6** and **9**), combination of 2,2′-bipyridine and N-(3-((4-nitrophenyl) thio)phenyl)methanediimine (complex **10)**, and (Z)-1-(4-morpholinophenyl)-N-((4-(trifluoromethyl) pyrimidin-2-yl)methyl)methanimine (complex **11)** (Radisavljević et al., 2018, 2019; Sankarganesh et al., 2019; Tabrizi et al., 2020), and primary biomolecules were examined by different experimental methods. These complexes were selected having in mind that the presence of different nitrogen-donor inert ligands in the structure of some gold(III) complexes have great potential to stabilize metal ion and improve its binding affinity toward biomolecules under physiological conditions (Radisavljević et al., 2019).

The study of potential anticancer activity of different transition metal ion complexes primarily considers their interactions with small biomolecules, such as amino acids, peptides, nucleosides, nucleotides, and further the interactions with DNA and proteins. In this respect, interactions with L-methionine (L-Met), L-histidine (L-His), guanine (Guo), guanosine-5′-monophosphate (5′-GMP), inosine (Ino), and inosine-5′-monophosphate (5′-IMP) are very important and must be emphasized. These interactions are dependent on redox properties, electrophilicity, and kinetic lability of complexes. Sometimes, reactivity of the nucleophiles indicates deactivation of complexes (Gukathasan et al., 2019). For example, the reduction possibility of L-glutathione is well-known. Its concentration in the cells is 10 mM (Williams et al., 2017), so the chosen complexes must avoid reduction before reaching to the target. In general, square-planar complexes undergo two pathways for substitution (Skibsted, 1986; Tobe and Burgess, 1999). The first is solvolytic, which includes formation of solvent-complex before substitution with nucleophile. The second is direct attack by the nucleophile. In order to clarify interactions, substitution reactions of gold(III) complexes **1**–**3** with Guo and 5′-GMP, and further with DNA, were followed by stopped-flow technique. For these experiments, concentration of ligand was always at least a 10-fold excess, so the reactions are followed under *pseudo*-first order. The substitution was studied in the presence of 150 mM NaCl to avoid solvolysis and provoke direct nucleophile attack. The obtained values for the rate constants are given in Table S1. Also, negative values of entropy of activation ΔS^≠^ confirmed the associative mode of substitution for all studied systems. In comparison, complex **1** has shown the best activity. This could be explained by the presence of methyl groups in the structure of complexes **2** and **3** which decrease the reactivity of complexes due to their positive inductive effect as well as steric hindrance during the formation of five-coordinated transition state (Hofmann et al., 2003; Jaganyi et al., 2004). Monofunctional gold(III) complexes with terpyridine or diethylenetriamine as inert ligands show similar mechanisms of substitution (Deković et al., 2012).

It is well-known that DNA is the main target for platinum(II) complexes, while some gold(III) complexes can cause direct DNA damage (Patel et al., 2013). Interactions between CT-DNA molecule and studied complexes were examined by different experimental methods (UV-Vis, fluorescence measurements, or viscosity). The UV-Vis method was used just to reveal the interaction between complexes and CT-DNA, but exact mode of binding could not have been predicted on this way. In the case of complexes **1**–**9** and **11**, different spectra were obtained after addition of CT-DNA (Figure S1). The values of intrinsic binding constants K_b_ (M^−1^) for complexes **1**–**9** were calculated and given in Table 1. This observation was the evidence for very good affinity for binding of complexes toward CT-DNA.

**Table 1:** The DNA-binding constants (*K*_b_) and Stern–Volmer constants (*K*_sv_) from EB–DNA fluorescence for complexes **1–9** and bovine serum albumin (BSA) constants and parameters (K_sv_, k_q_, K, and n) derived for complexes **4**–**9** (Radisavljević et al., 2018, 2019).

**Complex**	**K_**b**_ [M^**−1**^]**	**K_**sv**_ [M^**−1**^]**	**K_**sv**_ [M^**−1**^]**	**k_**q[**_M^**−1**^ s^**−1**^]**	**K [M^**−1**^]**	**n**
**1**	(5.7 ± 0.1) × 10^3^	(1.6 ± 0.1) × 10^4^	(6.71 ± 0.07) × 10^4^	(6.71 ± 0.07) × 10^12^	(2.05 ± 0.04) × 10^4^	0.6
**2**	(4.6 ± 0.1) × 10^3^	(4.0 ± 0.1) × 10^3^				
**3**	(1.6 ± 0.1) × 10^3^	(3.0 ± 0.1) × 10^4^				
**4**	(3.72 ± 0.04) × 10^3^	(3.54 ± 0.04) × 10^4^				
**5**	(1.91 ± 0.06) × 10^4^	(4.01 ± 0.05) × 10^4^	(8.04 ± 0.08) × 10^4^	(8.04 ± 0.08) × 10^12^	(5.82 ± 0.06) × 10^4^	0.45
**6**	(1.69 ± 0.05) × 10^4^	(1.65 ± 0.02) × 10^4^	(4.41 ± 0.05) × 10^4^	(4.41 ± 0.05) × 10^12^	(1.84 ± 0.06) × 10^4^	0.64
**7**	(7.50 ± 0.02) × 10^3^	(6.339 ± 0.004) × 10^4^	(1.320 ± 0.004) × 10^5^	(1.320 ± 0.004) × 10^13^	(3.87 ± 0.04) × 10^4^	0.65
**8**	(9.85 ± 0.03) × 10^3^	(1.956 ± 0.002) × 10^4^	(7.131 ± 0.005) × 10^4^	(7.131 ± 0.005) × 10^12^	(1.98 ± 0.02) × 10^4^	0.59
**9**	(1.56 ± 0.05) × 10^4^	(4.783 ± 0.002) × 10^4^	(1.289 ± 0.008) × 10^5^	(1.289 ± 0.008) × 10^13^	(6.02 ± 0.05) × 10^4^	0.82

The same conclusion was derived for structurally similar complexes with terpyridine ligands (Liu et al., 1995; Messori et al., 2005; Shi et al., 2006). The other method, fluorescence spectroscopy, was employed in the aim to clarify the mode of interaction between complexes and DNA. For fluorescence measurements, besides DNA, classical intercalator ethidium-bromide (EB) was involved. The changes in the spectra of complexes **1**–**9** and **11** indicated good possibility of complexes to replace EB from EB-DNA adduct and bound to DNA *via* intercalation (Figure S2).

To confirm this mode of binding, viscosity measurements were done as well. The viscosity of DNA is dependent on the length changes and can clarify the intercalative binding (Li et al., 2010). After addition of complexes **1**–**9** or **11** to CT-DNA solution, the increasing of viscosity was explained by elongation of DNA helix due to the insertion of complex between base pairs (Figure S3).

The transportation of metal ions in the bloodstream may be affected by the high affinity of them for aminoacidic residues of plasma proteins, in particular serum albumin. Besides, serum albumins have impact on various physiological functions. They affect colloid osmotic blood pressure, and they are in charge for constant blood pH (He and Carter, 1992). Some previous research evidence worth antioxidant activity of albumins as well as the protection of oxidative stress (Bourdon et al., 1999). Serum albumins are general ligands for hematin, fatty acids, and bilirubin. Therefore, they can bind metal ions or complexes (Bal et al., 1998; Marcon et al., 2003) as well. The domains of serum albumin are I, II, and III, and each of these has subdomains A and B. Subdomains IIA and IIIA are free for binding of ligands or complexes and their other names are Sudlow site I and site II (Carter et al., 1989; Carter and Ho, 1994). The differences between human serum albumin (HSA) and BSA are number of amino acid residues (585 for HSA; 582 for BSA), molecular weight (66.5 kDa for HSA; 66 kDa for BSA) and number of tryptophan (Trp) residues (Trp at position 214 for HSA; Trp at positions 213 and 134 for BSA) (Paul et al., 2019). Beside these differences, the high structural homology makes BSA a suitable model in these investigations. Interactions between gold(III) complexes and BSA were followed by fluorescence spectroscopy and for complexes **4**–**9** formation of BSA–gold(III) compound was confirmed (Figure S4). BSA-binding constants (K) and other parameters, such as K_sv_, Stern-Volmer quenching constant; k_q_, quenching constant; K, BSA-binding constant; and n, number of binding sites per albumin, derived for complexes **4**–**9** are given in Table 1. Accordingly, during the experiments were not mentioned the reduction of gold(III) complexes upon interaction with protein, respectively.

## Cytotoxic Activity of Gold(III) Complexes

The cytotoxicity and selectivity of different transition metal ion complexes on various cancer cells play the key role in the design and development of new potential anticancer agents. At the beginning of the twenty-first century, gold(III) porphyrin complex, with satisfying physiological stability, was reported like promising anticancer agent with IC_50_ values in the range of 0.73–0.11 μM. The studies against normal and cisplatin-resistant ovarian cancer have shown the ability of gold(III) porphyrin complex, *in vitro* and *in vivo*, to exceed cisplatin resistance without affecting health tissue (Lum et al., 2014; Bauer et al., 2019). Furthermore, carbamate-based Au(III) complexes were used for animal experiments and based on the observed data of clinical studies gave promising results of good anticancer effects (Marzano et al., 2011; Gu et al., 2019). All these facts have contributed to the assumption that gold(III) complexes have different modes of action compared with platinum antitumor active compounds (Nobili et al., 2009).

The potential activity of **1**–**11** was estimated by 3-(4,5-dimethythiazol-. 2-yl)-2,5-diphenyl tetrazolium bromide (MTT) test on different cell lines (A549, A375, and LS-174 for complex **1**, 4T1 and CT26 for complexes **1**–**3**, MDA-MB-231, HCT-116 for complexes **1**–**9**, MRC-5 for complexes **4**–**9**, MCF-7 for complexes **10**–**11**, HT-29 for complex **10**, HepG2 and NHDF for complex **11**, HeLa for complexes **1**–**3** and **10**–**11**). The results are summarized in Table S2.

Complexes **1**–**3** have shown better antitumor effects than cisplatin in the case of both breast cancer cell lines (MDA-MB-231 or 4T1). Cytotoxicity against CT26 was weaker than against HCT116 and much lower for than for cisplatin. Consequently, results have shown the reduction of the amount of antiapoptotic protein Bcl-2 in the presence of complexes **1**–**3**, but there was not emphasized significant reduction in the activation of Bax protein. Complexes **1**–**3** had a possibility to increase the amount of capsase-3 compared to untreated cells (Zarić et al., 2020). These results confirm a different influence of bulky inert tridentate nitrogen-donor ligand on activity toward different cell lines. The promising IC_50_ values for complex **1** led to the further examination of this complex. Investigation of cell cycle phase distribution in A375 cells after addition of complex **1** indicated the interaction with DNA. Results of apoptotic potential of complex **1** showed increasing of the percentage of cells in early as well as in late apoptosis. The treatment of A375 cells, first with N-acetyl-L-cysteine and after with complex **1**, suggested impact on reactive oxygen species (ROS) level and antiproliferative effects. After 3 h of cell treatment, decrease in ROS level was noticed, which pointed out the possibility of complex to interfere level of ROS in A375 cells. Complex **10** indicated generation of ROS in a stronger way than cisplatin. ROS and mitochondria are very important to explain apoptosis induction by gold(III) complexes in the case of cancer (Živanović et al., 2017).

For complex **10**, notable cytotoxicity was noticed against HT-29 and MCF-7 cancer cell lines in comparison with cisplatin, while for HeLa cancer cells, activity was the same like for cisplatin (Tabrizi et al., 2020). The results of cytotoxicity against MRC-5 (non-tumorigenic cells) for complex **10** were much higher than for cisplatin. All examined complexes showed significant cytotoxicity compared with cisplatin (Petrović et al., 2015) or with some other platinum(IV) complexes (Stojković et al., 2014) and palladium(II) complexes (Altaf et al., 2017).

For complexes **4**–**9** cytotoxic effects were noticed (IC_50_ < 100 μM) as well. After 1 day of treatments, these results were significant, in comparison with K[AuCl_4_] (Table S2). Generally, complexes **7**–**9** have shown better cytotoxicity. Namely, the longer diamine chain as well as the number of diamines led to the increasing of hydrophobic properties and makes gold(III) complexes more appropriate and flexible for entrance in the cells. The highest activity of complexes **4**–**9** was remarked for colorectal cancer cells (HCT-116).

## Computational Calculations

The highest occupied molecular orbital (HOMO) and lowest unoccupied molecular orbital (LUMO) present the useful tool which can be used to clarify chemical reactivity as well as stability of compounds (Solomon et al., 2012, 2014). DFT calculations for complexes **4**–**9** show that they possess distorted square-planar coordination geometry. HOMOs for dinuclear complexes were concentrated on one gold center, while for trinuclear complexes, LUMOs were concentrated on the central gold(III) ion.

Computational calculations are very powerful methods for prediction or explanation of DNA/BSA interactions with complexes, binding places, and binding affinity (Warren et al., 2006). Based on the results obtained by molecular docking, differences in ligand structures (aromatic or aliphatic) were emphasized (Figure S5). For these experiments, 1BNA (DNA structure dodecamer) was used due to the fact that interactions with 1BNA can reflect interactions with DNA. Docking analysis for complexes **1**–**3** and **11** showed interaction *via* intercalation, while complexes **4**–**9** fitted in the minor groove on DNA. Results for complexes **1**–**3** have shown that only complex **1** can form H-bonds because of hydrogen atoms (Figure 1). The presence of these H-bonds had great impact on stabilization of complex **1**–DNA product. The weakest interaction between complex **3** and DNA was confirmed due to the intercalation between base pairs which is not complete. Explanation for this is steric hindrance for complex **3** which led to negative value of binding energy. In the case of complexes **4**–**9**, the presence of diamine length allowed better interaction with DNA. Also, docking results indicated that complexes **4**–**9** can be bound to BSA to subdomain IIA (site I) forming hydrogen bonds, while electrostatic and hydrophobic interactions had the major impact on binding (Radisavljević et al., 2019). For complex **11** was proved the presence of hydrogen bonds with Arg185, Leu189, Thr190, Ser192, Pro240, Glu424, Ser428, Ile552, and Arg458 on the active site of BSA (Sankarganesh et al., 2019).

## Conclusion

We described here the characteristics of some newly synthesized gold(III) complexes as well as their interactions with important biomolecules and DNA/BSA by different experimental methods and by theoretical calculations. The results of the substitution reactions show a good affinity of complexes **1**–**3** toward different biomolecules, such as Guo, 5′-GMP, and DNA. Presented results have indicated the good possibility for interaction between gold(III) complexes and DNA/BSA, with high values of binding constants. Interactions with DNA are confirmed with constants in the range between 10^3^ and 10^4^ (M^−1^) determined by UV-Vis and fluorescence measurements, respectively, while the calculated constants for BSA interactions are in the range between 10^4^ and 10^5^ (M^−1^). All results were supported with computational calculations, and clear explanations of the reactions with DNA/BSA were obtained by molecular docking studies. Biological investigations of studied gold(III) complexes toward different cell lines emphasized their potential compared with cisplatin or K[AuCl_4_]. All observed results could be very useful for further investigation of potential anticancer drugs, having in mind that a lot of scientists every day make efforts to find new transition metal complexes with better activity and selectivity than cisplatin.

## Author Contributions

SR wrote the manuscript. BP supervised the manuscript.

## Conflict of Interest

The authors declare that the research was conducted in the absence of any commercial or financial relationships that could be construed as a potential conflict of interest.
