# Supplementary material for: Gold(III) Complexes: An Overview on Their Kinetics, Interactions With DNA/BSA, Cytotoxic Activity, and Computational Calculations
Source: Front Chem. 2020 May 20;8:379. doi: 10.3389/fchem.2020.00379 (PMC7251155; doi:10.3389/fchem.2020.00379)
Supplement: Supplementary file 1 [file Data_Sheet_1.docx]

Supplementary Material

**Gold(III) complexes: An overview on their kinetics, interactions with DNA/BSA, cytotoxic activity and computational calculations**

**Snežana Radisavljević^1^, Biljana Petrović^2^**^*^

^1^Department of Chemistry, Faculty of Science, University of Kragujevac, Radoja Domanovića 12,
P.O. Box 60, 34000 Kragujevac, Serbia

**Table S1.** Rate constants and activation parameters for the substitution reactions between complexes **1 – 3** and Guo, 5'-GMP or DNA (Radisavljević et al., 2018).

|  | **T [K]** | **10^3^ × k_2_[M^-1^ s^-1^]** | **k_1_ [M^-1^ s^-1^]** | **ΔH ^≠^ [kJ mol^-1^]** | **ΔS^≠^ [J^-1^k mol^-1^]** |
| --- | --- | --- | --- | --- | --- |
| **1**  *5’GMP* | 298 | 6.8 ± 0.2 | 0.12 ± 0.05 | 6.0 ± 0.7 | -170 ± 3 |
| *Guo* | 298 | 8.7 ± 0.4 | 1.4 ± 0.7 |  |  |
| *DNA* | 298 | 9.6 ± 0.3 | 1.5 ± 0.6 |  |  |
| **2** |  |  |  |  |  |
| *5’-GMP* | 298 | 5.3 ± 0.3 | 0.8 ± 0.2 | 17 ± 3 | -132 ± 8 |
| *Guo* | 298 | 7.2 ± 0.3 | 0.3 ± 0.01 |  |  |
| *DNA* | 298 | 7.7 ± 0.3 | 0.7 ± 0.2 |  |  |
| **3** |  |  |  |  |  |
| *5’-GMP* | 298 | 4.4 ± 0.2 | 0.9 ± 0.4 | 20 ± 2 | -126 ± 6 |
| *Guo* | 298 | 7.0 ± 0.4 | 0.2 ± 0.02 |  |  |
| *DNA* | 298 | 7.3 ± 0.4 | 0.6 ± 0.1 |  |  |

**Table S2.** Cytotoxic effects - IC_50_ values (μM) of gold(III) complexes (**1** - **11**), K[AuCl_4_] and cisplatin (Radisavljević et al., 2018; Radisavljević et al., 2019; Sankarganesh et al., 2019; Tabrizi et al., 2020, Zarić et al., 2020).

| **Complex** |  |  |  | IC_50_ | | | | | |  |  |  |  |
| --- | --- | --- | --- | --- | --- | --- | --- | --- | --- | --- | --- | --- | --- |
|  | LS-174 | A549 | A375 | MDA-МB-231 | | HCT-116 | | MRC-5 | | HeLa | MCF-7 | HT-29 | HepG2 |
|  |  |  |  | 24h | 72h | 24h | 72h | 24h | 72h |  |  |  |  |
| **1**  **2**  **3** | 11.2 | 20.1 | 13.1 |  | 1.6  15.1  5.4 |  | 0.7  53.9  4.1 |  |  | 1.3  3.4  5.7 |  |  |  |
| **4** |  |  |  | 95.86 | 89.37 | 72.62 | 29.88 | 57.36 | 62.88 |  |  |  |  |
| **5** |  |  |  | 88.84 | 52.83 | 56.5 | 52.03 | 58.51 | 57.72 |  |  |  |  |
| **6** |  |  |  | 77.07 | 58.54 | 60.17 | 37.16 | 34.23 | 53.27 |  |  |  |  |
| **7** |  |  |  | 98.17 | 75.05 | 0.25 | 15.88 | 51.78 | 89.23 |  |  |  |  |
| **8** |  |  |  | 93.92 | 52.78 | 23.28 | 24.18 | 28.37 | 29.97 |  |  |  |  |
| **9** |  |  |  | 98.07 | 61.67 | 63.34 | 133.87 | 63.81 | 83.10 |  |  |  |  |
| **10** |  |  |  |  |  |  |  |  | 30.17 | 3.09 | 2.05 | 0.65 |  |
| **11** |  | 33.19 |  |  |  |  |  |  |  | 32.00 | 20.6 |  | 22.68 |
| **K[AuCl_4_]** |  |  |  | 101.29 | 150.73 | >200 | 180.36 | 230.49 | 107.39 |  |  |  |  |
| **cisplatin** | 24.4 | 24.1 | 23.1 |  | 30.8 |  | 1.6 |  |  | 26.7 |  |  |  |


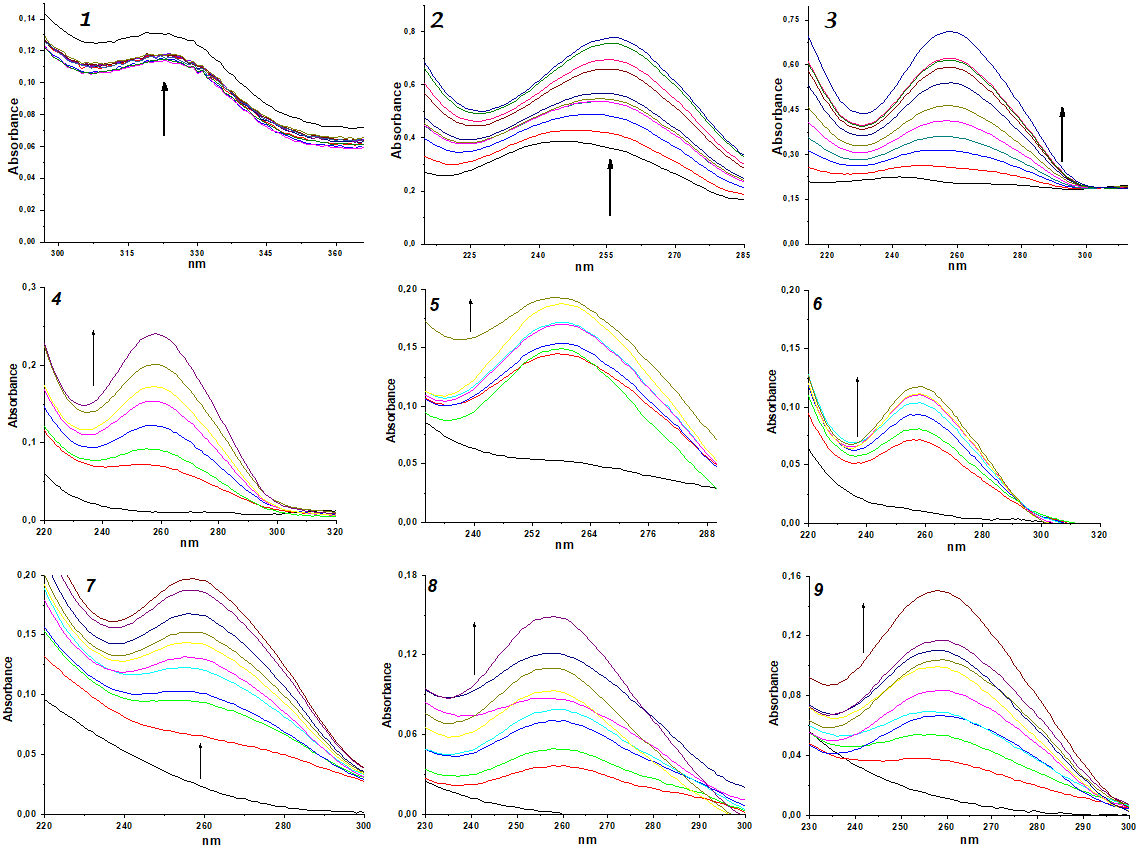


**Figure S1.** Absorption spectra of the complexes **1 – 3** in 10 mM Tris-HCl/150 mM NaCl pH = 7.4, upon addition of CT-DNA. [complex] = 1.35 x 10^-5^ M, [CT-DNA] = (1.35-13.5) x 10^-5^ M. Absorption spectra of complexes **4** - **9** in 0.01 M PBS (pH=7.4) upon addition of
CT-DNA, [complex] = 8 × 10^-6^ M, [CT-DNA] = (0 – 3.84) × 10^-5^ M. Arrow shows the absorbance changes upon increasing the DNA concentration.


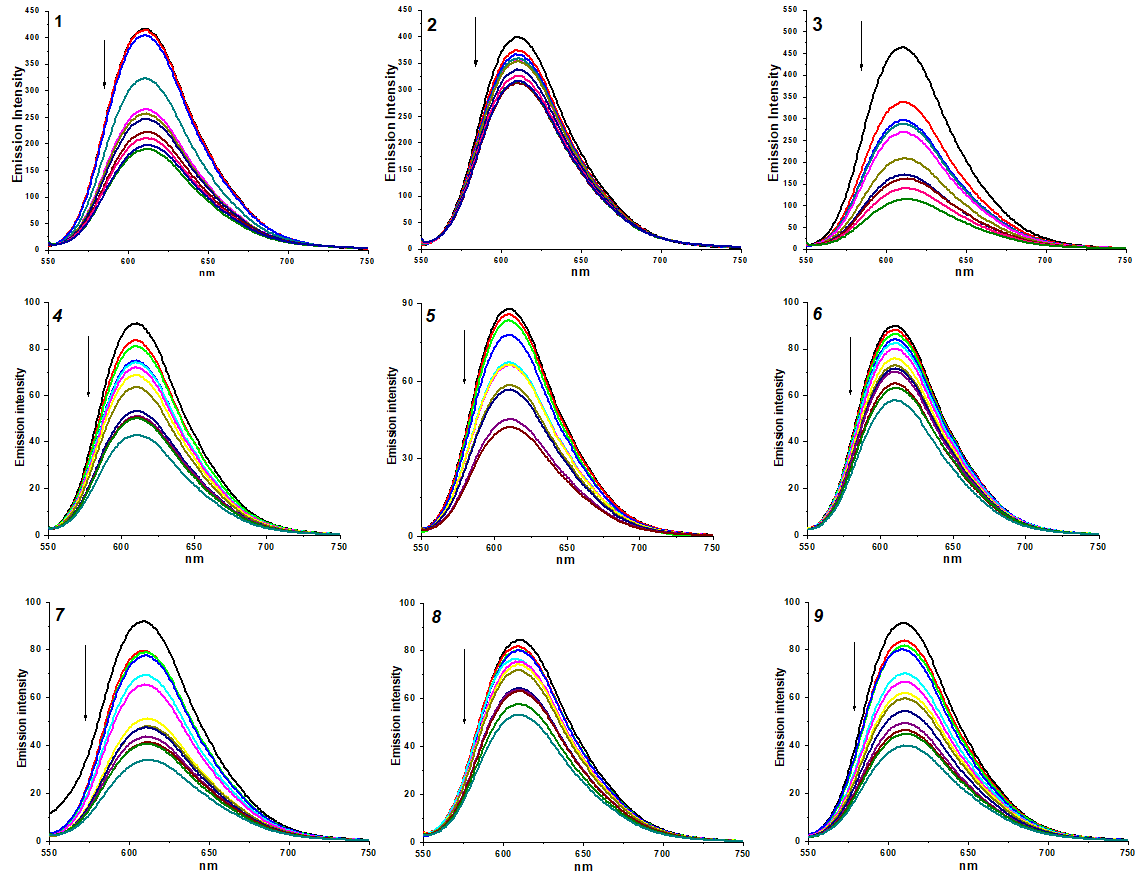


**Figure S2.** Emission spectra of EB bound to DNA in the presence of complexes **1**– **3**, [EB] = 80 μM, [DNA] = 80 μM; [complex] = 0–80 μM; *λ*_ex_= 527 nm, *λ*_em_= 612nm;
[EB] = 21 μM, [DNK] = 21 μM, [complex] = 0 – 29.4 μM for complexes **4 - 6** and [EB] = 18 μM,
[DNK] = 18 μM, [complex] = 0 – 25.7 μM for complexes **7 - 9**; Arrows show the intensity changes upon increasing the concentration of complex.


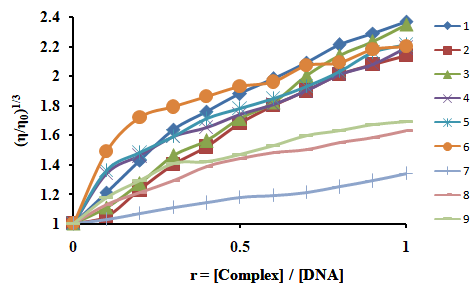


**Figure S3.** Relative viscosity (ɳ/ɳ_o_)^1/3^ of CT-DNA (0.01 mM) in 10 mM Tris-HCl/150 mM NaCl, pH=7.4 in the absence/presence of the increasing amounts of complexes **1**– **3** (r), for complexes
**4** – **9** in PBS buffer solution (pH=7.4) and concentration of CT-DNA 12 μM.


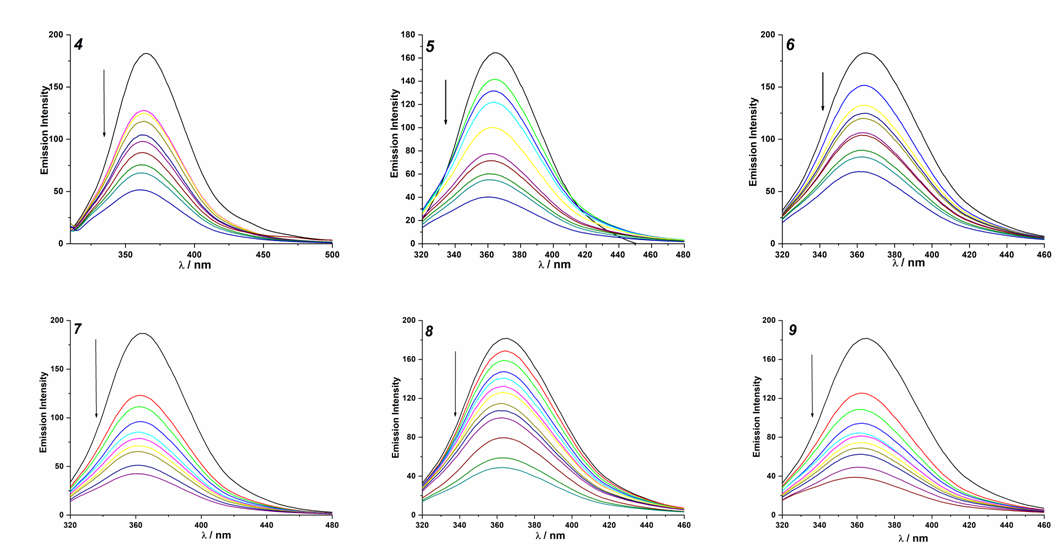


**Figure S4.** Emission spectra of BSA in the presence of complexes **4 – 9**, [BSA] = 2 μM, [complex] = 0 – 40 μM; λ_ex_= 295 nm. The arrows show the intensity changes upon increasing the concentration of complex. Insert: plots of I_0_/I *vs.* [Q].


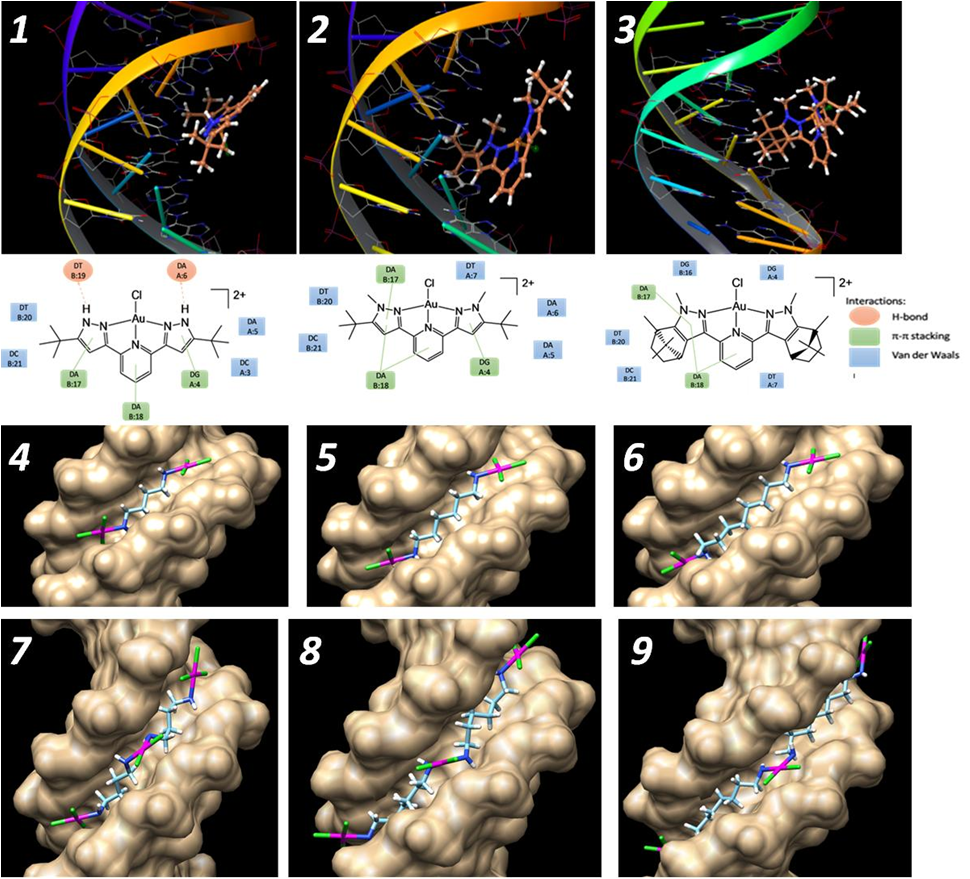


**Figure S5.** Visual representation of complexes **1 – 3** bound to 1BNA dodecamer and computational docking model illustrating interactions between complexes **4 – 9** and DNA.
